# Clinician-Scientists in Canada: Barriers to Career Entry and Progress

**DOI:** 10.1371/journal.pone.0013168

**Published:** 2010-10-04

**Authors:** Bryn Lander, Gillian E. Hanley, Janet Atkinson-Grosjean

**Affiliations:** 1 Centre for Health Services and Policy Research, School of Population and Public Health, University of British Columbia, Vancouver, British Columbia, Canada; 2 W. Maurice Young Centre for Applied Ethics, College for Interdisciplinary Studies, University of British Columbia, Vancouver, British Columbia, Canada; Medical Research Council South Africa, South Africa

## Abstract

**Background:**

Clinician-scientists play an important role in translating between research and clinical practice. Significant concerns about a decline in their numbers have been raised. Potential barriers for career entry and progress are explored in this study.

**Methods:**

Case-study research methods were used to identify barriers perceived by clinician-scientists and their research teams in two Canadian laboratories. These perceptions were then compared against statistical analysis of data from Canadian Institutes of Health Research (CIHR) databases on grant and award performance of clinician-scientists and non-clinical PhDs for fiscal years 2000 to 2008.

**Results:**

Three main barriers were identified through qualitative analysis: research training, research salaries, and research grants. We then looked for evidence of these barriers in the Canada-wide statistical dataset for our study period. Clinician-scientists had a small but statistically significant higher mean number of degrees (3.3) than non-clinical scientists (3.2), potentially confirming the perception of longer training times. But evidence of the other two barriers was equivocal. For example, while *overall* growth in salary awards was minimal, awards to clinician-scientists increased by 45% compared to 6.3% for non-clinical PhDs. Similarly, in terms of research funding, awards to clinician-scientists increased by more than 25% compared with 5% for non-clinical PhDs. However, clinician-scientist-led grants funded under CIHR's Clinical thematic area decreased significantly from 61% to 51% (p-value<0.001) suggesting that clinician-scientists may be shifting their attention to other research domains.

**Conclusion:**

While clinician-scientists continue to perceive barriers to career entry and progress, quantitative results suggest improvements over the last decade. Clinician-scientists are awarded an increasing proportion of CIHR research grants and salary awards. Given the translational importance of this group, however, it may be prudent to adopt specific policy and funding incentives to ensure the ongoing viability of the career path.

## Introduction

A gap exists between health research and clinical practice. While scientists constantly produce new knowledge relevant to clinical care, much of their work is never incorporated into practice [1], [2]. Similarly, clinical problems are rarely translated into research projects [3]. Clinician-scientists play an important role in filling this gap because they split their time and interests between clinical practice and research, enabling them to translate their research results into the clinic and to develop research questions based on clinical issues they encounter in practice [4]–[7]. Thus, much attention has been paid to the role of the clinician-scientist in research translation. Significant concern has been expressed over the last three decades about barriers faced by clinician-scientists and indeed their very survival. Both within Canada and the United States reports and articles on clinician-scientists point to a decreasing workforce, a lack of research grants, and clinician-scientists moving away from patient-based research towards basic science or health services research [5], [7]–[12].

In Canada, reports of a significant drop in the percentage of grantees with an MD or MD/PhD from Canada's major medical funding agency at the time, the Medical Research Council (MRC), began receiving attention over a decade ago [5]. However, the reform of Canada's medical research funding in 2000, which transformed the MRC into the Canadian Institutes of Health Research (CIHR), was accompanied by substantial reinvestments in Canadian health research [13]. Despite these reforms, the viability of patient-based research by clinician-scientists continues to be questioned [14]. It has been suggested that there are fewer clinician-scientists conducting research in Canada today than before the reforms. There have also been reports that clinician-scientists are turning away from the CIHR's Clinical thematic research area towards the other three foundational domains: Biomedical; Health Systems and Services; and Population and Public Health. Such reports argue that this shift makes clinician-scientists less likely to capitalize on their ability to conduct research relevant to the clinic and translate this research into clinical practice [10], [14].

Canadian physician and hospital care is covered by a universal public health insurance program which pays doctors primarily through fee-for-service mechanisms. Universities are also publicly funded with operating costs predominately provided on a provincial basis and research predominately funded through federal grant agencies such as CIHR. Canada has eighteen medical schools that are affiliated with hospitals. Many Canadian clinician-scientists work within these affiliated hospitals and may fulfill the three social missions of teaching, research, and care. Other clinician-scientists follow different career paths. Some clinician-scientists have faculty positions, others rely on grants and clinical practice for their salaries and may not be involved in teaching; some clinician-scientists devote the majority of their time to research, others are predominately focused on clinical care [8].

Given the importance of clinician-scientists to the development of clinically relevant research questions and the translation of these findings into practice, we felt it was important to understand whether, following the advent of CIHR, Canadian clinician-scientists continue to face barriers to career entry and progress. Due to a lack of high-quality data on the qualifications and activities of CIHR investigators, past studies tended to extrapolate clinician-scientist research trends from funding allocations among the four thematic areas. However, using a dataset that includes specific information on the training background of all CIHR grantees between 2000 and 2008, we were able to identify all Canadian clinician-scientists receiving CIHR support, regardless of thematic area. Furthermore, past articles—written as commentary and perspective pieces on the status of clinician-scientists—tended to use *either* qualitative data from clinician-scientists [9] *or* quantitative data on training and grants [5], [7]. We believe it is important to combine these two styles, using “mixed methods” to generate a more nuanced understanding of the real and perceived barriers faced by clinician-scientists in Canada. We adopted qualitative methods to understand the barriers perceived by particular clinician-scientists and their research teams and quantitative methods to explore whether these perceived barriers were supported in CIHR data.

## Methods

Ethics approval for the qualitative case study was granted by the Behavioural Research Ethics Board at the University of British Columbia. All participants signed a consent form. Ethics approval for the quantitative analysis was not required because it was covered by the publicly available data clause (Item 1.3.1) of the University of British Columbia's Policy #89: Research and other studies involving human subjects.

### Qualitative Methods

The research reported on here derives from a nested case study situated within a larger case study exploring the translational activities of a network (6 nodes; 120 members) of clinician-scientists, bioinformaticians, and basic scientists studying the pathogenomics of innate immunity. (The PG-II Network.) Case studies are concentrated enquiries investigating a particular bounded entity or phenomenon. Studies of particular cases help advance understanding of more general phenomena but are not, by their nature, statistically generalizable. The nested case described in this article focused on the two laboratories (LabA and LabB) in the node that provided PG-II with a clinical interface. LabA and LabB are located in a research institute on the campus of an urban referral hospital. The clinician-scientists heading the laboratories spend approximately 80% of their time on research and the remaining 20% as immunological specialists in the adjacent hospital. The purpose of the case was to identify the translational practices mediating the clinical and research goals of these particular clinician-scientists and their laboratories.

Qualitative data collection spanned February 2007 through January 2008. First, face-to-face surveys were administered to all 16 scientists and technicians employed in the two laboratories at the time of our visit. Subsequently, following analysis of the survey data, in-depth semi-structured interviews were conducted with the 10 remaining members of the original sample–six graduate and co-op students had, by this time, been rotated-out and replaced. Survey and interview questions explored issues such as the translational activities pursued within the lab; the role of funding agencies in supporting the work; training experiences and roles within the lab; and collaborations with other research groups. As well, between May and August 2007, a period of participant observation allowed us to monitor the formal and informal mechanisms influencing the work of the laboratories, and increased both the diversity of our data and our confidence in it. In the follow-up phase (January 2009), we reinterviewed four specific members of the team, selected on the basis of their expertise and engagement in the translational areas we had chosen for closer study: chronic granulomatous disease; IRAK-4 deficiency; and the longstanding bacteria-identification service provided by LabA to clinicians treating cystic fibrosis patients. Field notes, open-ended survey responses, and interview transcripts were coded with Atlas.ti qualitative data analysis software; we used SPSS for statistical analysis of survey data.

Our qualitative coding matrix captured information on funding, knowledge production, research culture, research values, training, and the translational interface. To ensure reliability in applying the matrix, one interview was separately coded by three members of the team. Variations in coding application were discussed and consistent definitions agreed upon. All transcripts were then coded by one team member.

### Quantitative Methods

To compensate for the case study's lack of generalizability, we accessed data from CIHR's Funded Research database to look for evidence of the barriers that clinician-scientists identified in the qualitative findings. This database is publicly available online and provides information on all funding granted by CIHR since its inception in 2000. The CIHR database contains fields indicating type of award (operating grant, training award, salary award, etc), principal investigators (PI), co-applicants (Coap), primary thematic areas, effective date of award, and monetary amounts funded for each type of award in each fiscal year. (For example, if a five year award with a total value of $500,000 was paid at a rate of $100,000 a year, our data would indicate the $100,000 separately for each fiscal year.) Through a special request to CIHR, we were also able to obtain information on the degrees obtained by each of the researchers in the dataset. These data indicate whether the researcher has a Bachelor's degree, a Master's degree, an MD, a doctoral degree in another health profession, a PhD, a fellowship, and whether they have completed a Postdoctorate or are a Registered Nurse. These fields are not mutually exclusive—researchers indicate all the degrees/training that they have completed.

Using the data provided by CIHR, we built a dataset including each unique investigator PIN, by degrees held, training and salary awards funded, and the funding Institute for each thematic domain of CIHR's mandate: (1) Biomedical; (2) Clinical; (3) Health Systems and Services; and (4) Population and Public Health. Categorization of research into the four themes is made by principal investigators when applying for grants. (See the CIHR glossary [http://www.cihr.ca/e/34190.html#r7] for details on how CIHR defines its four themes.) We calculated the total amount of funding for grants in each of the four themes, as well as the total amount of funding for training and salary awards across the nine fiscal years of data we had accessed (00/01–08/09). In the figures, we present both numbers of awards and dollars awarded as all grants are not equal. Canadian dollars are used throughout.

We defined clinician-scientists broadly to include those (including MDs and RNs) without a PhD and those with a PhD (such as MD-PhDs or RN-PhDs). We then compared this group to non-clinical scientists with PhDs in order to examine the perceived barriers we had identified. This broad definition allowed us to investigate the hypothesis that those trained as clinician-scientists may be moving away from patient-based research in the Clinical theme in favour of other CIHR thematic areas. Our definition is also consistent with that used in similar previous studies [5], [7], allowing for greater comparability.

Data were summarized using descriptive statistics. We performed statistical analyses to examine whether population means were significantly different using Chi-squared tests for categorical data and T-tests for normally distributed continuous variables. Statistical significance was determined at P-value less than 0.05. All analyses were performed using Stata version 10.0 (StataCorp, College Station, TX).

## Results

### Perceived barriers identified in our qualitative research

A key theme that emerged during data analysis was that clinician-scientists, and their laboratory colleagues, perceive barriers unique to their work. While we use representative quotes from the two principal clinician-scientists to outline these barriers, our interviews with laboratory colleagues and technicians supported their observations and added depth to our understanding. These lab workers saw firsthand, and were often affected by, the barriers encountered by their principals. The clinician-scientists we interviewed perceived high entry and maintenance barriers to their chosen career path that would discourage new entrants.

If you look at historical trends, clinician-scientists are a dying breed…the hard thing is to convince somebody to take this on as a career. It's hard enough to get funded as a basic scientist who doesn't have any clinical responsibilities. But to continue to run a large research lab and have clinical responsibilities is a daunting double life. A lot of people just aren't prepared to take that on.

Beyond the general challenges referenced in the above quote, three major perceived barriers were identified from our field data, related to (1) research training, (2) research salaries, and (3) research funding.

#### Research training

It is often the case that clinician-scientists complete medical training before starting research training. As a result, just at the point when they can expect to realize significant clinical earnings, and when many already carry sizeable student loans, potential clinician-scientists must again defer financial rewards. Our sources suggest that this combination of earnings deferral and prolonged training acts as a significant disincentive for prospective new recruits: “the major discouragement is poverty and a very long training program.”

#### Research salaries

Clinical faculty often earn significantly more than research faculty. This is a problem for clinician-scientists since universities tend to be reluctant to pay the clinical rate for research work. As one clinician-scientist noted: “the university doesn't want to pay a research scientist much, much more [just] because they have a medical degree.” Thus, for a research team, finding money to pay clinician-scientists is often the most challenging part of hiring them. One solution, according to our sources, is to put together the equivalent of a clinician's salary on an *ad hoc* basis from multiple research and clinical “pockets,” with CIHR as the deepest pocket in the research mix. These salary sources are often temporary and arbitrary, however, with no guarantees of continuity. Informants argue that formal payment plans need to be established for clinician-scientists if the career path is to be a viable option.

#### Research funding

The playing-field for research funding is viewed as far from level. Clinician-scientists, who spend part of their time in patient-based practice, find themselves competing with full-time research scientists. Funding applications are judged on the research experience of the applicant; clinical experience is not an important factor in these decisions. As one of our informants noted:

You have to submit grants that will be fundable at the national and international level, and papers that will be published in internationally recognized journals. And the journals or granting agencies don't care much [about the clinical part].

Thus, clinician-scientists felt they were less likely to accumulate grants (and the publications essential to funding success) than researchers with no clinical responsibilities whose focus was tied intimately to the terms and conditions of the granting agencies. In the words of one informant, “I'm competing with basic scientists; I'm looking for the same CIHR dollars as somebody who has no clinical responsibilities.” An administrator who helps scientists improve their funding applications commented:

[For] a clinician-scientist who sees a problem in a patient and then brings that into the lab…you have to be more creative as to where you're [looking for] funding because it fits outside of the box.

He notes that review criteria tend to focus on factors like number and quality of publications, numbers of trainees graduated. “[But] number of clinical guidelines changed? [That doesn't count.]”

### Are these perceived barriers borne out in the CIHR data?

#### Research Training

The clinician-scientists in our dataset (n = 4,522) did have a small but statistically significant higher mean number of degrees than their non-clinical PhD counterparts (n = 8,346) (3.3 versus 3.2 p-value <0.005). Combined MD/PhD training programs represent an attempt to overcome the disincentives of extended training by integrating the clinical and research components. To address the problem of reduced earning potential, CIHR has instituted specific clinician-scientist training awards designed to support qualified clinicians interested in pursuing a research career. The ‘Clinician Scientist Phase 1’ award provides a training stipend for up to 6 years, while the ‘Clinician Scientist Phase 2’ award is restricted to holders of Phase 1 awards, providing up to 6 years of salary support once training is completed.

Our data indicate that CIHR funded a total of 127 MD/PhD studentships between 00/01 and 08/09. The total amount spent on these studentships was $10.1 million. However, over 70% were awarded between 00/01 and 04/05, meaning that a smaller number of awards have been made in the past four fiscal years. With respect to the ‘Clinician Scientist Phase 1 award’, 87 award winners received support over the study period at a mean annual value of $43 thousand; there was no shift apparent in the number of annual awards. During the same time frame, a total of 60 “Clinical Scientist Phase 2” awards were granted worth a total of $12.8 million with a mean annual value of $20.5 thousand.

#### Research salaries

CIHR funding for salary awards remained relatively stable, increasing from approximately $29 million in 00/01 to $31 million in 08/09. Clinician-scientists received approximately 34% of the salary awards (32% of total dollars), while non-clinical PhDs received approximately 63% of the awards (64% of total dollars.) While overall funding for salary awards held fairly stable, clinician-scientists had the largest growth rates in the category, increasing by a mean of 45.5% compared to a mean of 6.3% for non-clinical PhDs. Salary award funding for other researchers decreased over the study period. The mean annual value of a CIHR-funded salary award was $53.6 thousand and there was no significant difference in mean or median value between clinician-scientists and non-clinical PhDs (p-value 0.11 and 0.32, respectively).

#### Research funding

**Figure 1:**
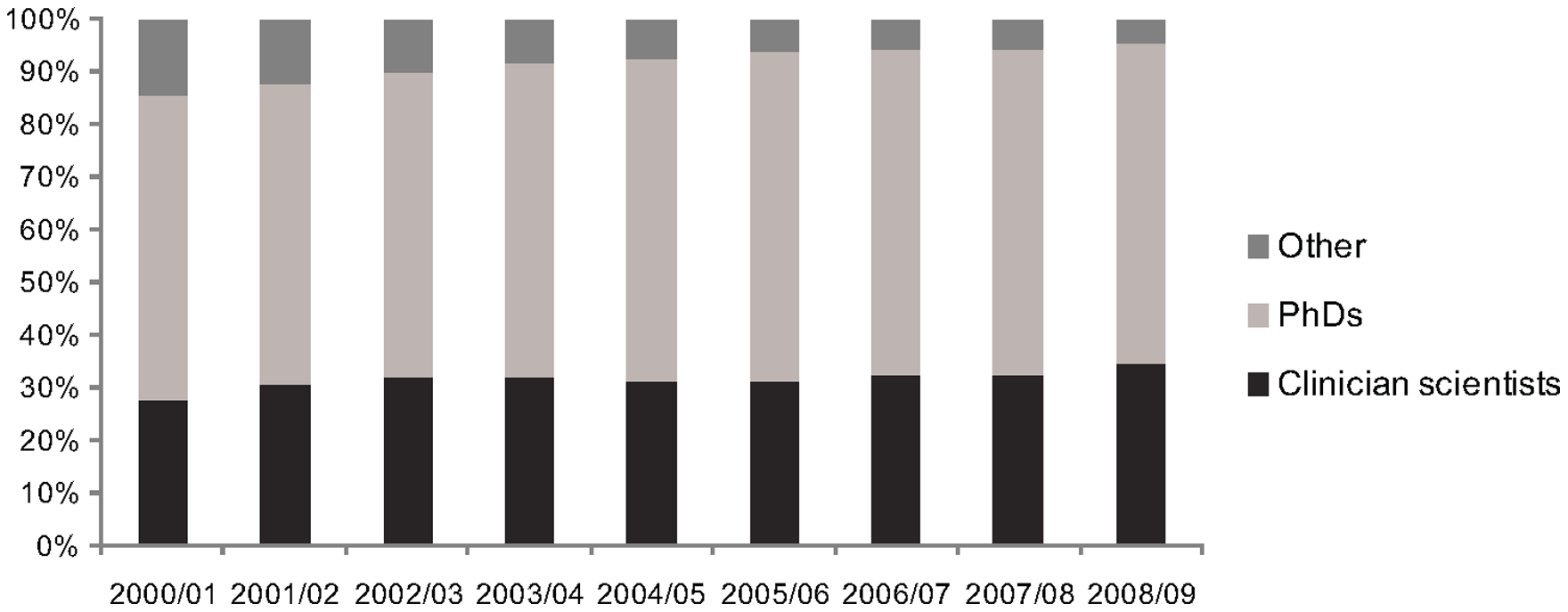
Funding to clinician-scientists, non-clinical PhDs, and other researchers between 2000/01 and 2008/09.

Figure 1 illustrates the overall percentage of total grant funding that went to clinician-scientists, non-clinical PhDs and other researchers by fiscal year. The figure indicates that funding to clinician-scientists grew slightly over the study period, from approximately 28% of total funding in fiscal year 00/01 to approximately 35% by 08/09. Over the same period, the growth rate in grant funding for non-clinical PhDs was 5% compared to 25% for clinician-scientists. The results are similar when number of grants awarded are used, rather than monetary values of the grants.

**Figure 2:**
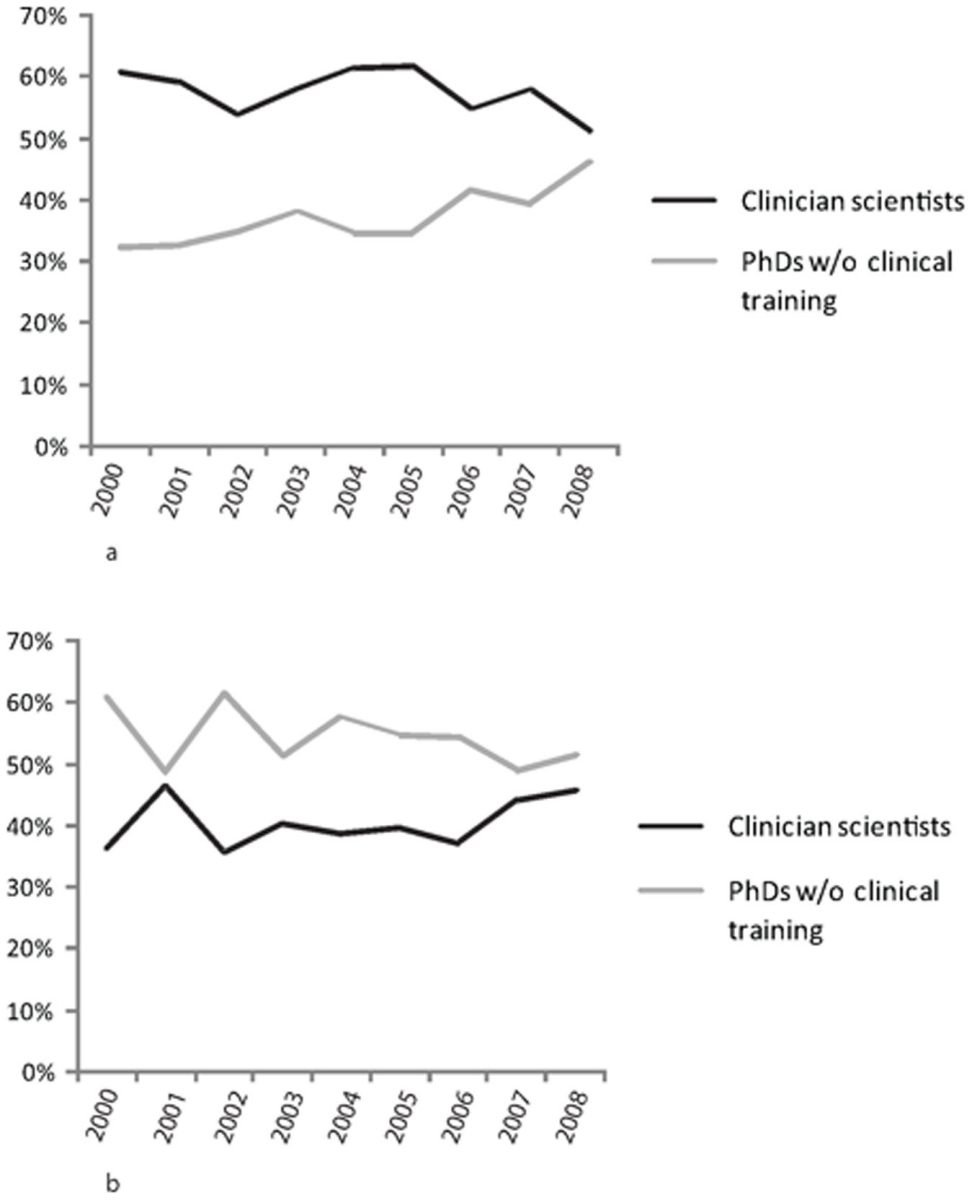
a: Percentage of clinical research grants led by clinician-scientists and non-clinical PhDs between 2000 and 2008. **b:** Percentage of health systems and service research grants led by clinician-scientists and non-clinical PhDs between 2000 and 2008.

To examine the hypothesis that clinician-scientists are moving away from CIHR's Clinical thematic domain in favour of other theme areas we examined the percentage of Clinical grants led by at least one clinician-scientist across the entire study period. Figure 2a indicates that the percentage decreased significantly from 61% in 2000 to 51% in 2008 (p-value <0.001). The number of non-clinical PhDs leading Clinical research grants increased from 32% to 46% in the same period (p-value <0.001). The percentage of Biomedical research grants led by clinician-scientists remained relatively stable while Population and Public Health grants grew slightly. However, grants under the Health Systems and Services thematic area increased significantly, from 36% in 2000 to 46% in 2008 (p-value <0.001) as indicated in Figure 2b.

**Figure 3:**
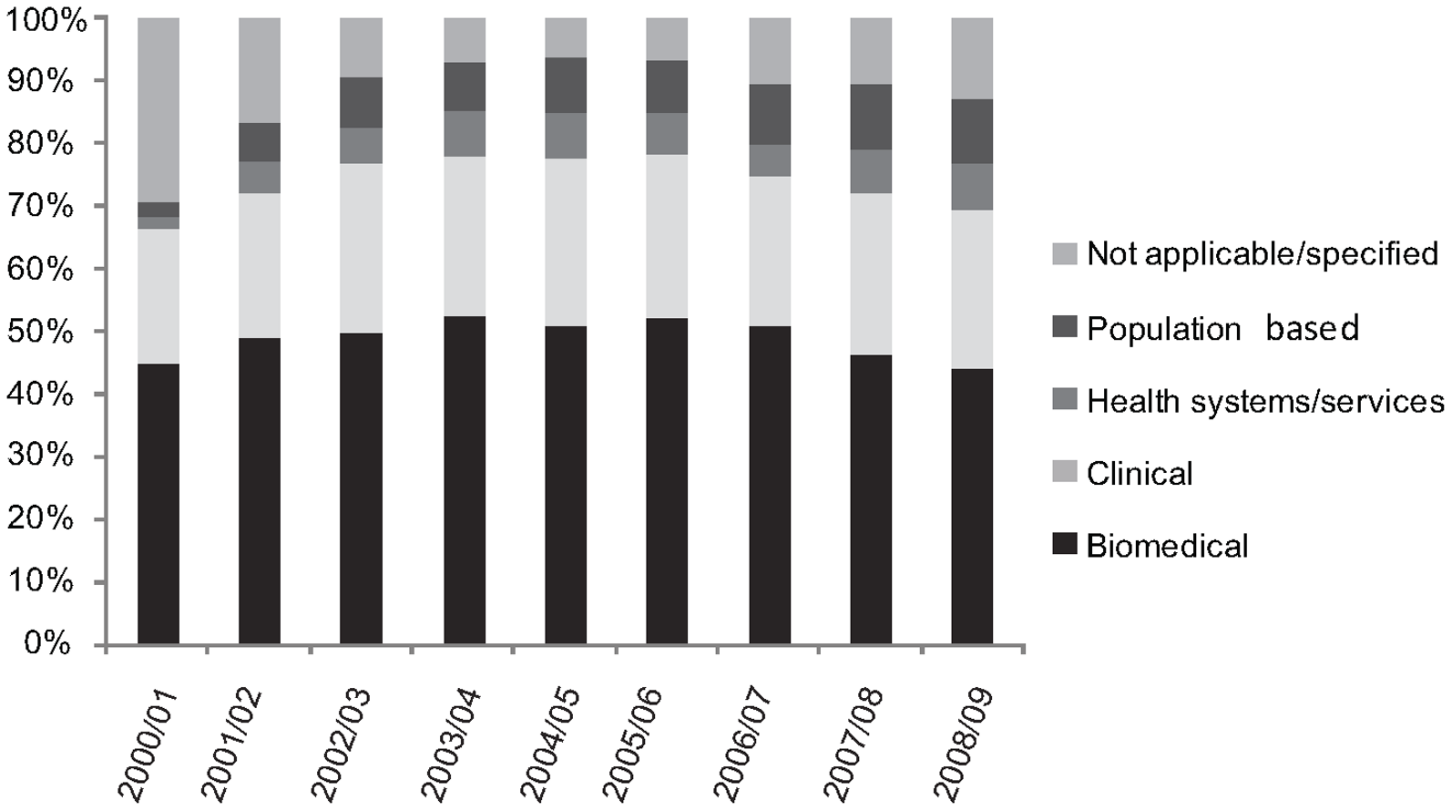
Thematic distribution of amount funded to clinician-scientists by CIHR research grant programs between 2000/01 and 2008/09.

Figure 3 shows the percentage of clinician-scientists' funding that comes from each of the CIHR thematic research areas. While they may be moving towards other areas, clinician-scientists still receive the majority of their funding from the Biomedical and Clinical research themes; an average of 74% of their total funding came from this combination.

## Discussion

Our research suggests that a tension may exist between barriers perceived by clinician-scientists and actual CIHR support for this career category. The quantitative data for the period (00/01 through 08/09) suggest considerable stability and some growth in research funding and training support for clinician-scientists. Differences between the qualitative and quantitative findings may be caused by a disconnect between the ‘lived experience’ of individual clinician-scientists and what we detected at a broader population level. For example, while our results show increasing salary support for clinician-scientists, no significant differences exist between median salary award amounts for clinician-scientists and non-clinician PhDs. This levelling does little to remedy the higher salaries paid to clinicians over scientists, a differential known to act as a disincentive for clinicians thinking about a research career [7], [8], [15].

The inconsistency between our qualitative and quantitative findings might also be explained by a lack of awareness about CIHR programs instituted to address the barriers, such as the MD/PhD studentships and the Clinician Scientist Phase 1 and 2 awards. Declines in clinician-scientist rates did occur in Canada in the 1980s and 1990s [5] and current perceptions may lag real increases in support. Also, we lack data on the success rate of applications to CIHR. A significantly lower success rate for clinician-scientists compared to non-clinical peers might explain why a barrier is perceived that is absent in the statistical data. Also, our quantitative analysis examined only one source of funding (CIHR) for clinician-scientists, excluding provincial funders (e.g. Michael Smith Foundation for Health Research, Alberta Heritage Foundation for Medical Research), disease organizations (e.g. Heart and Stroke Foundation, Canadian Cystic Fibrosis Foundation), as well as industry. While our analysis does not paint a full picture of all funding sources in the country, CIHR is recognized to be the largest single funder of health research grants in Canada [16]. Finally, this inconsistency between our qualitative and quantitative findings might be explained by the particularity of case-study research methods. Our nested case was bounded by the larger case of the PG-II network. In other words, our study was confined to those clinician-scientist led laboratories *within* PG-II. As a result, it involved colleagues working within the same discipline, at the same organisation, and within the same research network. While this tight focus provided a satisfying level of detail and richness, we recognize that the barriers we identified may be specific to the discipline or organisation. A study employing comparative case methods might well produce different results. Our results are consistent with Roy who noted a drop in clinician-scientist grantees from over 40% to close to 33% between 1986 and 1995 [5]. Beginning in 00/01, we found that clinician-scientists were collecting only 28% of CIHR grant money; however, this percentage increased to 35% by 08/09. These results suggest that the situation of clinician-scientists might have worsened before beginning to recover. While our quantitative analysis looked at trends over a nine year period, our qualitative data explored barriers perceived by clinician-scientists today. Their perception is that while barriers may have been lowered since CIHR was founded, they still exist; this perception mirrors some of the trends we found in the quantitative data.

Rosenberg argues that the bridging role played by clinician-scientists in the United States sets the United States apart from Japan and Europe [9]. This would also set Canada apart from Japan and Europe. Consistent with previous findings for Canada [5], in the United States clinician-scientist trainee and grant applications to the National Institutes of Health (NIH) declined between 1971 and 1978 [7], a trend which continued into the 1990s [9]. Again as in previous Canadian findings [10], [14], the proportion of clinician-scientists involved in clinical research in the United States fell from 40% of NIH applications in 1972 to 25% in 1997 [17]. Similar to recent CIHR initiatives in Canada, between 1998 and 2002, a series of programs initiated by the NIH and non-for-profit institutions focused on encouraging clinician-scientist training [18]. While the number of clinician-scientists conducting research remained flat into the early 2000s, there are signs that more are entering the career pipeline [19]. Thus, the cautious optimism shown in our results appears consistent with recent trends within the United States. In England, clinician-scientists appear to have received less attention [20] and we were unable to locate comparable data. Instead, British policies appear more focused on encouraging bridges between basic research and clinical practice more generally as opposed to supporting clinician-scientists specifically. Policy initiatives encouraging such bridges date to the 1971 Rothschild Report [21] and more recently through the creation of biomedical research centres that bring England's public health system and universities together [22].

The quantitative results of this study seem to paint a more optimistic picture about the role of clinician-scientists in Canada's research enterprise than previous studies. Nevertheless, we found that clinician-scientists and their trainees still perceive important barriers to working in this area—barriers they believe act as fundamental disincentives towards pursuing careers as clinician-scientists. Our data show that while some progress has been made on some barriers, less progress has been made on others. Many government programs and policies have been implemented to address the impediments but these have been piecemeal initiatives rather than an overarching strategy. Individuals wishing a career as a clinician-scientist can pursue an integrated MD/PhD program, short term research training, or longer full term training. Subsequent salaries are often created organization by organization on an ad hoc basis through a combination of clinical fee schedules and research dollars [8].

In order to ease some of the barriers perceived by clinician-scientists, our qualitative findings suggest that funding agencies need to develop a method for evaluating research proposals that does not penalise clinical work [8], [17]. Connections to the clinic provide a niche advantage for clinician-scientists and this area should be emphasized in research policy and planning. Continuing to broaden research training and salary award programs and creating formal salary policies on an organizational level would likely support continued growth in numbers of clinician-scientists.

This study represents an attempt to create a more nuanced understanding of barriers perceived by clinician-scientists since the advent of CIHR. As such, it represents a first of many potential steps to further understand the issue. A qualitative study of clinician-scientists involved in different disciplines and thematic areas would help to show whether perceived barriers are similar or different across the board. A longitudinal study would help to track how perceptions change over time. Analyses of institutional (university and hospital) policies on clinician-scientists would help identify structural barriers. A quantitative study that develops a dataset capable of including funding from disease organisations and industry as well as CIHR, would paint a more comprehensive picture of where clinician-scientists obtain their support.

Clinician-scientists play a vital role in translating between clinical practice and discovery research. We believe their role needs to be better understood and appreciated in a policy and funding climate that promotes translational practices [23].
